# Functional and Clinical Outcomes in Acute Wound Management: Measuring the Impact of Negative Pressure Wound Therapy and Specialized Physical Therapy

**DOI:** 10.3390/life15040511

**Published:** 2025-03-21

**Authors:** Cristina-Teodora Stanciu, Dinu Vermesan, Daniel Laurentiu Pop, Bogdan Hogea, Silviu Valentin Vlad

**Affiliations:** 1 Doctoral School, “Victor Babes” University of Medicine and Pharmacy Timisoara, 300041 Timisoara, Romania; cristina.stanciu@umft.ro; 2 Department XVI—Orthopedics, Traumatology, Urology, and Medical Imaging, Discipline of Orthopedics and Traumatology I, “Victor Babes” University of Medicine and Pharmacy Timisoara, 300041 Timisoara, Romania; dinu@vermesan.ro (D.V.); daniellaurentiupop@yahoo.com (D.L.P.); 3 Department of Surgery, Faculty of Medicine, University of Oradea, 410073 Oradea, Romania; siviu.vlad@didactic.uoradea.ro

**Keywords:** negative pressure wound therapy, rehabilitation techniques, proprioceptive neuromuscular facilitation, wound healing, joint mobility

## Abstract

Background and Objectives: Optimizing functional recovery alongside wound healing remains a challenge in acute wound management. Negative Pressure Wound Therapy (NPWT) is widely used to promote tissue regeneration and reduce edema, yet its impact on functional outcomes and quality of life is less explored. This study evaluates the effects of NPWT alone versus NPWT combined with physiotherapy, focusing on functional recovery and patient-reported outcomes. Materials and Methods: This prospective study included patients with acute wounds at the Timisoara County Emergency Clinical Hospital, treated between 2020 and 2024. Participants were divided into two groups: Group 1, receiving NPWT exclusively, and Group 2, undergoing NPWT combined with physiotherapy (Proprioceptive Neuro-muscular Facilitation, Kabat diagonals, manual lymphatic drainage, and proprioceptive exercises). Assessments included joint mobility (goniometry), edema (circumferential measurements), muscle strength (Manual Muscle Testing), and patient-reported outcomes using WHOQOL-BREF, SF-36, and HADS questionnaires. Results: Results demonstrated that, at 10 days, patients in the specialized physiotherapy group had significantly greater ankle dorsiflexion (18.10 ± 1.63°) compared to the classical group (10.05 ± 1.76°; *p* < 0.001). Knee flexion in the specialized group was 134.58 ± 5.15° versus 115.57 ± 5.32° in the classical group (*p* < 0.001). Edema circumference and depth were reduced in both groups, with minor but notable improvements in the specialized group at later follow-ups (*p* < 0.05). Self-reported quality of life (SF-36, WHOQOL-BREF) and mental health (HADS) scores were slightly better at 10 days in the specialized group, although differences diminished by 6 months. Conclusions: Combining NPWT with specialized physiotherapy techniques enhances functional recovery and quality of life in acute wound patients. These findings support the integration of multi-disciplinary rehabilitation to optimize patient outcomes.

## 1. Introduction

Acute wounds, often resulting from severe trauma or complex surgical interventions, continue to pose a significant challenge in modern medical practice. The healing process of such wounds is dynamic and influenced by a range of local, systemic, and therapeutic factors, underscoring the need for personalized and integrated approaches to wound management [1,2]. Acute wounds demand not only effective local wound care but also a comprehensive understanding of the systemic factors that influence healing outcomes [1]. Beyond addressing the immediate wound, therapeutic interventions should also consider the functional consequences of acute wounds, such as restricted joint mobility, persistent edema, and muscle strength deficits. These complications can prolong recovery and diminish patients’ autonomy.

Negative Pressure Wound Therapy (NPWT) is recognized as a standard technique in the treatment of acute wounds due to its ability to reduce edema, stimulate tissue regeneration, and accelerate wound healing [3,4]. NPWT significantly accelerates the healing process by enhancing granulation tissue formation and reducing bacterial colonization [3]. However, the exclusive use of NPWT does not directly address the functional impairments that may arise from acute wounds. This limitation highlights the potential role of physiotherapeutic interventions in complementing NPWT by targeting mobility restoration, edema reduction, and muscle strength recovery.

Despite notable advances in wound management, there is limited research examining the combination of NPWT and physiotherapy techniques. Recent studies have begun to explore this combination, suggesting potential benefits in functional recovery [5]. Most existing studies focus either on the isolated effects of NPWT on tissue healing or on the general benefits of physiotherapy in rehabilitation, without exploring the potential synergy between these interventions [6,7,8,9]. Furthermore, there is a lack of comprehensive data regarding the impact of combining these therapies on clinical and functional outcomes, such as joint range of motion, muscle strength, and the reduction in post-traumatic complications.

The aim of this study is to compare the efficacy of NPWT used alone versus NPWT combined with physiotherapeutic interventions, focusing on functional and clinical parameters in patients with acute wounds. By contributing to a better understanding of integrated therapeutic strategies, this research aims to highlight the potential of combined interventions to improve clinical outcomes and accelerate functional recovery.

## 2. Materials and Methods

### 2.1. Study Design and Settings

This study was designed as prospective research, conducted between September 2020 and September 2024 at the Clinical County Emergency Hospital of Timisoara, Department of Orthopedics-Traumatology I. This period allowed for the inclusion of a representative number of patients with acute wounds and medium-term monitoring of progress regarding functional and clinical parameters.

Demographic, clinical, and functional data were collected through a secure electronic database accessible only to authorized personnel, ensuring the protection of confidential information in accordance with international regulations (EU GCP Directive 2005/28/EC) and national legislation (Law no. 95/2006). The study protocol was approved by the hospital’s ethics committee, and all patients provided informed consent prior to inclusion in the study.

The first group represented patients treated exclusively with NPWT, focusing on the effectiveness of this standard method in optimizing the healing process.

The second group represented patients treated with NPWT combined with specialized physiotherapy techniques, using an integrated approach to optimize functional outcomes. This protocol included techniques such as Proprioceptive Neuromuscular Facilitation (PNF), Kabat diagonals, manual lymphatic drainage, and proprioceptive exercises.

The interventions for Group 2 were structured into daily sessions lasting 45–60 min, conducted during hospitalization. After discharge, patients followed a personalized outpatient program tailored to their individual progress. While NPWT accelerates wound healing, combining it with functional rehabilitation techniques can significantly enhance patient outcomes in terms of mobility and strength recovery [3].

The study design was structured to allow a rigorous comparative evaluation of functional and clinical parameters between the two groups, using validated methods for objective measurements such as joint mobility, muscle strength, and edema volume. Through this design, the study provides valuable insights into the efficiency of integrated interventions and contributes to the development of optimized therapeutic strategies for patients with acute wounds.

### 2.2. Inclusion and Exclusion Criteria

The criteria applied in this study were developed to select a homogeneous and clinically relevant population to obtain valid and reproducible results. The study included patients with acute wounds treated with NPWT, localized in the lower limbs, specifically in the calf area. These patients were monitored throughout hospitalization and within a structured functional recovery program. Inclusion criteria: (1) patients aged between 19 and 66 years; (2) injuries localized in the calf area, without severe complications (open tibial fractures, post-surgical wounds, or crush injuries), treated with NPWT; (3) provision of informed consent to participate in the study; and (4) willingness of patients to undergo all proposed therapeutic interventions, including the functional recovery program applied to Group 2. No other primary treatment was offered to patients during the study.

Exclusion criteria: (1) patients with other types of wounds, such as diabetic ulcers, pressure ulcers, or extensive necrotic lesions; (2) presence of significant comorbidities that could influence the healing process; (3) refusal to sign the informed consent form or failure to attend scheduled periodic evaluations; (4) non-participation of patients in the functional recovery program (for Group 2); and (5) the occurrence of severe complications during the study that could affect final results. All included participants were evaluated at key moments using standardized tools for collecting clinical and functional data. The exclusion of cases that did not meet established criteria was carried out in accordance with the protocol.

All patients were screened and enrolled by a team of attending orthopedic surgeons and wound care specialists who assessed each injury using standardized clinical guidelines for acute wounds. Specifically, wounds were classified as acute if they had occurred within a 3-week period following recent trauma or surgery and showed no clinical indicators of chronic inflammation or prolonged healing delays. This evaluation ensured that participants’ wounds remained within the acute phase, aligning with the study’s focus on early-stage wound healing and rehabilitation.

Patients were assigned to the two study groups by a research coordinator who applied a computer-generated randomization sequence in sealed, opaque envelopes. This approach minimized selection bias and ensured that neither the attending clinicians nor the patients could predict group allocation, preserving the integrity of the randomization process.

Documentation for each case was conducted in line with international guidelines, using the ICD-10 classification system to ensure data uniformity and comparability [8]. Leduc et al. emphasized the importance of “standardized data collection methods and classification systems like ICD-10 to ensure consistency and comparability in clinical studies” [9]. The rigorous selection of patients was essential for achieving the study’s objectives and obtaining a homogeneous and representative population.

### 2.3. Data Collection and Questionnaires

This study focused on collecting and analyzing clinical and functional variables to examine the impact of combining NPWT with specialized functional rehabilitation techniques on acute wound healing and patient quality of life. Demographic data, including age, gender, body mass index (BMI), lifestyle habits (smoking, alcohol consumption), marital status, and residential environment (urban or rural), were obtained from electronic medical records and supplemented through direct observations and evaluations conducted during interventions [1]. Data confidentiality was ensured according to existing regulations, with access restricted solely to medical personnel involved in the research, as stipulated in the EU GCP Directive 2005/28/EC [2].

Clinical and functional evaluations focused on parameters such as edema, joint range of motion (ROM), and muscle strength, documented at regular intervals [3]. The ROM was measured using a goniometer for the ankle and knee joints, while edema was assessed via circumferential measurements using a flexible tape [4]. Muscle strength was determined through the application of standardized Manual Muscle Testing (MMT) protocol.

Wound size was quantified by measuring length and width in centimeters at consistent reference points around each injury site, ensuring comparability across different anatomical locations. A sterile ruler or disposable measuring tape was used to record these dimensions, which were then multiplied to approximate the wound’s surface area. Depth was additionally assessed in cases of complex wounds using a sterile probe or cotton swab to ensure accurate tracking of overall wound progression.

To assess patients’ quality of life, two internationally validated questionnaires were employed: the WHOQOL-BREF, which offers a comprehensive evaluation of general health and quality of life [10], and the SF-36 Health Survey, which measures both physical and emotional health domains [11]. These questionnaires were administered at three distinct time points: at the initiation of the study, at six weeks, and at the end of the follow-up period, providing a detailed perspective on patient progress.

Patients’ mental health status was investigated using the Hospital Anxiety and Depression Scale (HADS), a validated instrument for assessing anxiety and depression levels in clinical settings [11]. All evaluations were conducted by qualified personnel using standardized equipment, and the resulting data were meticulously documented to ensure high-quality statistical analysis. Results were reported as means and standard deviations, ensuring clarity and statistical relevance in the conclusions drawn from the study [12].

### 2.4. Statistical Analysis

The analysis of the collected data was performed using SPSS statistical software, version 26.0 (IBM Corp., Armonk, NY, USA) [13]. A priori power analysis was conducted using G*Power (version 3.1, Heinrich-Heine-Universität Düsseldorf, Düsseldorf, Germany) to determine the necessary sample size for detecting a medium effect size (Cohen’s *d* = 0.50) in the primary outcome measure (e.g., range of motion). The calculation assumed a two-tailed test with an alpha level of 0.05 and a power of 80%. Results indicated that each group required at least 70 participants to reliably detect the hypothesized between-group difference. To accommodate potential dropouts and incomplete data, we inflated this estimate by approximately 20%, arriving at a target total of 168 participants.

Continuous variables were expressed as mean ± standard deviation (SD), while categorical variables were presented as frequencies and percentages. For comparing the means of continuous variables between the two study groups, Student’s *t*-test was applied. Categorical variables were analyzed using the chi-square test to determine statistical associations.

To evaluate the association between patient-reported outcomes (e.g., WHOQOL-BREF and HADS) and objective measurements (e.g., joint mobility, muscle strength, edema reduction), Pearson and Spearman correlation analyses were applied, depending on data distribution. This approach allowed the investigation of the impact of combining Negative Pressure Wound Therapy with specialized physiotherapy techniques on the physical, mental, and social dimensions of quality of life.

The statistical significance threshold was set at *p* < 0.05. Advanced statistical methods, such as the Barnard test and the Wang interval, were considered to enhance the robustness of analyses, especially given the study’s sample size. To minimize the risk of Type I errors resulting from multiple comparisons, the Bonferroni correction was applied. In addition to reporting *p*-values, we also calculated Cohen’s *d* (for continuous variables) and Cramér’s V (for categorical comparisons) to complement the standard significance testing.

## 3. Results

### 3.1. Patient Demographics

A total of 203 patients were initially enrolled in the study according to selection criteria, but 11 patients were excluded due to loss at follow-up after treatment. Therefore, the final sample consisted of 192 eligible patients. Group 1 included 110 patients treated exclusively with NPWT, while Group 2 consisted of 82 patients who received NPWT combined with specialized physiotherapy techniques. The average age of patients was 37.86 years in Group 1 and 36.54 years in Group 2. Gender distribution was similar between the two groups, with a slight predominance of males (*p* > 0.05).

Regarding the types of wounds treated, 37.3% of patients in Group 1 presented with open tibial fractures, 42.7 had post-surgical wounds (osteosynthesis or external fixations), and 20% suffered from crush injuries. In Group 2, the proportions were similar: 32.9% had open tibial fractures, 50% had post-surgical wounds, and 17.1% experienced crush injuries. The differences between groups in wound type distribution were not statistically significant (*p* = 0.688), as seen in Table 1.

### 3.2. Evolution of Functional Parameters

**Figure 1:**
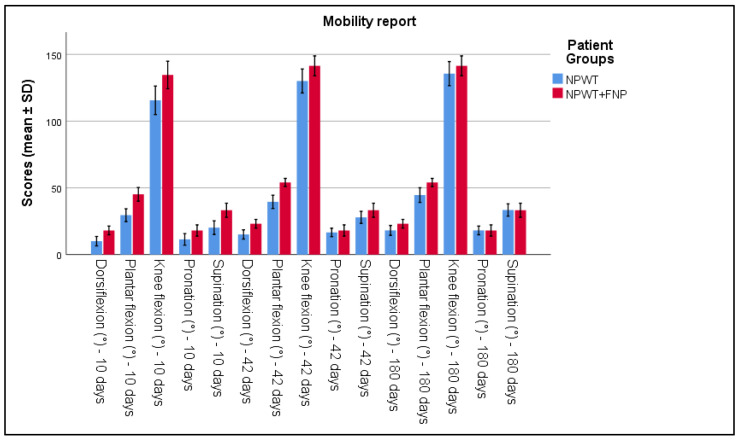
Analysis of the mobility report over the 3 periods of time.

The evaluation of functional and clinical parameters highlighted significant differences between the two patient groups. Joint mobility, measured using a standardized goniometer, showed notable improvements in Group 2. The range of motion at the ankle increased significantly by the tenth day of treatment, compared to Group 1 (*p* < 0.05). Similarly, knee mobility improved quicker in Group 2 versus Group 1. This can be seen in Table 2 and Figure 1.

**Figure 2:**
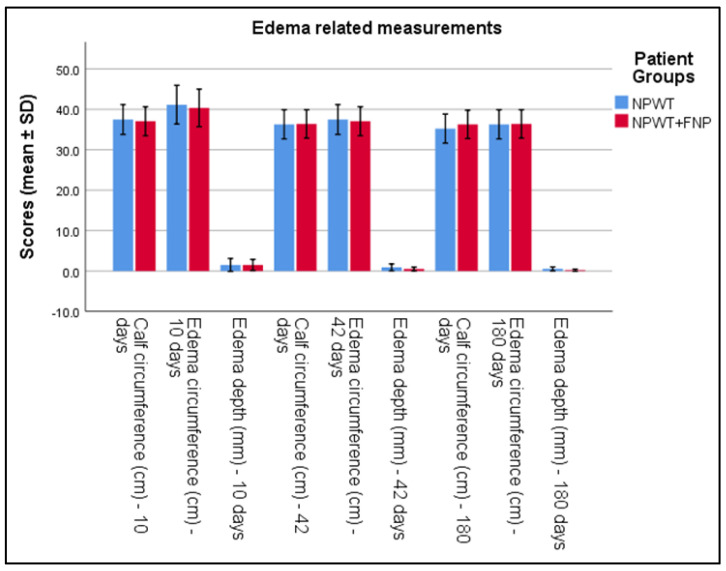
Analysis of the calf, edema circumference, and edema depth measurements over the 3 periods of time.

Table 3 presents significant findings in edema management between two groups treated with Negative Pressure Wound Therapy (NPWT) exclusively and NPWT combined with specialized physiotherapy (NPWT+FNP). By the 10th day, edema circumference showed a significant reduction in the NPWT+FNP group (40.36 ± 2.32 cm) compared to the NPWT group (41.15 ± 2.39 cm), with a *p*-value of 0.023 and a Cohen’s d of 0.33. At 42 days, a notable decrease in edema depth was observed in the NPWT+FNP group (0.50 ± 0.23 mm) compared to the NPWT group (0.9 ± 0.42 mm), achieving statistical significance with a *p*-value of less than 0.001 and a Cohen’s d of 1.21. The long-term measurements at 180 days further highlighted significant differences; calf circumference was significantly smaller in the NPWT group (35.23 ± 1.79 cm) compared to the NPWT+FNP group (36.28 ± 1.76 cm), with a *p*-value of less than 0.001 and a Cohen’s d of 0.59. Additionally, edema depth significantly reduced to 0.19 ± 0.14 mm in the NPWT+FNP group versus 0.53 ± 0.24 mm in the NPWT group, also with a *p*-value of less than 0.001 and a Cohen’s d of 1.69. These measurements indicate significant improvements in edema management in patients receiving combined therapy compared to those receiving NPWT alone at specific time intervals, as seen in Table 3 and Figure 2.

**Figure 3:**
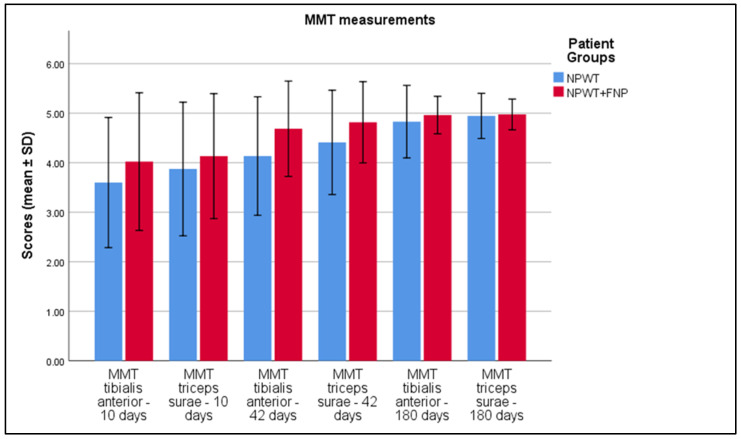
Analysis of the MMT results over the 3 periods of time.

Significant improvements were observed in the MMT scores at various time points. Initially, the MMT score for triceps surae was significantly higher in the NPWT group (3.46 ± 0.67) compared to the NPWT+FNP group (3.18 ± 0.64), with a *p*-value of 0.004 and a Cohen’s d of 0.43. By 10 days, both tibialis anterior and triceps surae showed significant improvements in the NPWT+FNP group, with scores of 4.02 ± 0.69 (*p* < 0.001, Cohen’s d = 0.63) and 4.13 ± 0.63 (*p* = 0.007, Cohen’s d = 0.38), respectively. At 42 days, further significant enhancements were recorded: tibialis anterior scores rose to 4.69 ± 0.48 in the NPWT+FNP group (*p* < 0.001, Cohen’s d = 1.02) and triceps surae rose to 4.82 ± 0.41 (*p* < 0.001, Cohen’s d = 0.86). At 180 days, the tibialis anterior score remained significantly higher in the NPWT+FNP group (4.96 ± 0.19) compared to the NPWT group (4.83 ± 0.37), with a *p*-value of 0.003 and a Cohen’s d of 0.48. There was no significant difference in the triceps surae scores at 180 days (*p* = 0.303), as presented in Table 4 and Figure 3.

### 3.3. Analysis of Quality of Life Questionnaires and Functional Parameters

**Figure 4:**
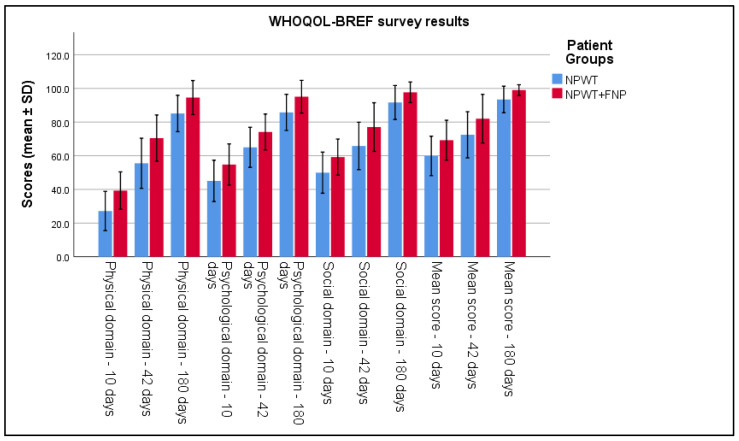
Analysis of the WHOQOL-BREF survey results over the 3 periods of time.

The WHOQOL-BREF questionnaires highlighted better functional recovery in Group 2. At 6 weeks post intervention, the average score in Group 2 was 82.02 ± 7.23, compared to 72.47 ± 6.86 in Group 1 (*p* < 0.001). The social domain also showed higher scores in Group 2 (*p* < 0.001), and mental health components increased by an average of 14% more compared to Group 1 (*p* < 0.001), indicating a significant improvement in psychological and emotional well-being, as seen in Table 5 and Figure 4.

**Figure 5:**
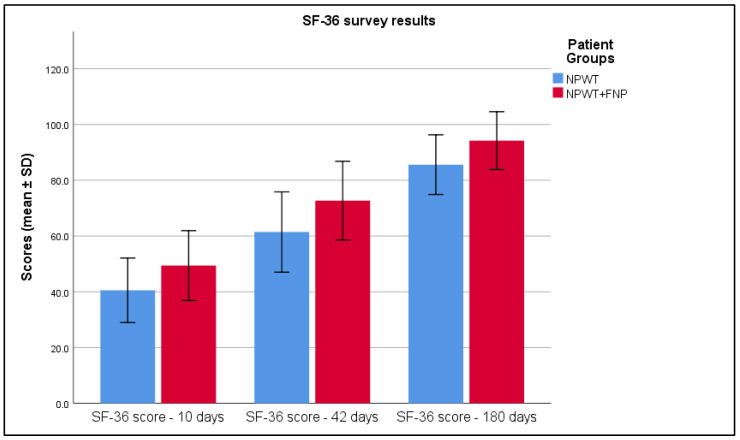
Analysis of the SF-36 survey results over the 3 periods of time.

The outcomes derived from the SF-36 questionnaire provided clear and robust confirmation of the observed trends. More specifically, the data indicated a notable and statistically significant increase in the measured values for Group 2 when compared to Group 1 (*p* < 0.001). This significant difference is comprehensively detailed in Table 6 and is visually emphasized in Figure 5, both of which illustrate the disparity between the two groups.

**Figure 6:**
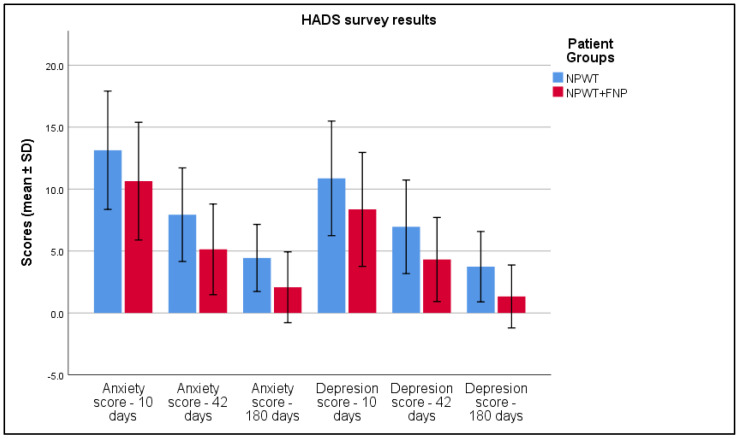
Analysis of the HADS survey results over the 3 periods of time.

Furthermore, a detailed analysis of scores from the Hospital Anxiety and Depression Scale (HADS) revealed lower anxiety values in Group 2 in week 6 (7.93 ± 1.89 versus 5.13 ± 1.83 in Group 1, *p* < 0.001) and a reduced depression score (6.95 ± 1.89 compared to 4.32 ± 1.70, *p* < 0.001), reflecting an improvement in mental health immediately after treatment, as seen in Table 7 and Figure 6.

**Figure 7:**
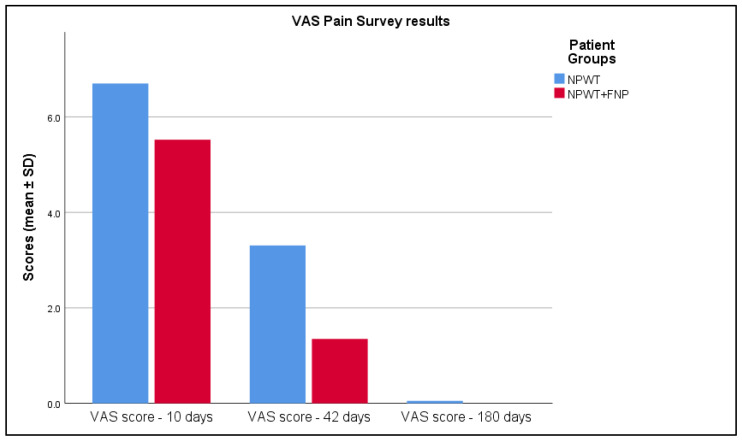
Analysis of the VAS-scale for pain survey results over the 3 periods of time.

The data in Table 8 and Figure 7 also reveal differences in pain reduction between the NPWT and NPWT+FNP treatment groups, assessed by the Visual Analog Scale (VAS) scores at various time points. At 10 days, Group 2 reported smaller pain levels with VAS scores of 5.52 ± 1.01, whereas Group 1 had 6.7 ± 1.05, with *p*< 0.001. However, by 42 days, the NPWT+FNP group experienced a greater reduction in pain, indicated by a lower VAS score of 1.35 ± 1.03 compared to 3.31 ± 1.34 in the NPWT group, with this difference being statistically significant (*p*< 0.001). At 180 days, both groups achieved a VAS score close to 0, with Group 1 having a score of 0.05 ± 0.31 whilst Group 2 presented 0 (*p* = 0.137), indicating no pain and showing complete pain alleviation in the long term.

Pearson and Spearman analyses demonstrated a significant correlation between improvements in joint mobility and increases in WHOQOL-BREF and SF-36 scores (*p* < 0.05), suggesting that objective motor function improvements are reflected in patients’ health perceptions. At 6 weeks post intervention, patients in Group 2 continued to show better scores for anxiety and depression (*p* < 0.05). By 10 weeks, although differences had decreased, Group 2 maintained a superior trend, indicating mental state stabilization in both groups, but with a persistent advantage for patients undergoing combined therapy (Table 9).

## 4. Discussion

### 4.1. Analysis of Literature

Our study highlights the significant benefits of integrating NPWT with specialized physiotherapy techniques on the recovery of patients with post-traumatic and post-surgical wounds, reinforcing prior findings [14,15]. The results showed significant differences between the two groups, both during hospitalization and throughout the 10-week follow-up. An additional evaluation at 6 months confirmed the persistence of benefits, particularly in Group 2, highlighting the long-term advantages of a multidisciplinary intervention.

From a clinical parameter perspective, Group 2 demonstrated better outcomes compared to Group 1. Edema reduction, measured through circumferential analysis, was also more pronounced in Group 2. Joint mobility also showed notable improvements: the range of motion increased for Group 2 compared to Group 1. Kisner and Colby noted that therapeutic exercises integrated with wound care protocols significantly enhance joint flexibility and functional restoration [2].

Regarding quality of life, assessed using the WHOQOL-BREF questionnaires, patients in Group 2 recorded an increase in the physical component compared to Group 1. At the 6-month re-evaluation, the difference between groups persisted, indicating that the functional progress achieved through interventions in Group 2 had long-term effects. These improvements can be attributed to both active mobilization and the positive influence of social interaction, characteristics associated with physiotherapy sessions. Leduc et al. supported this by stating that active rehabilitation not only accelerates physical recovery but also enhances social and emotional reintegration [9].

In terms of mental health, HADS scores showed lower levels of anxiety and depression in Group 2. At discharge, patients in this group had an average anxiety score of 10.64 ± 2.38 and a depression score of 8.36 ± 2.30, while Group 1 recorded anxiety and depression scores of 13.14 ± 2.39 and 10.87 ± 2.31, respectively (*p* < 0.001). Differences were maintained throughout the monitoring period, including after 10 weeks, confirming the favorable influence of physiotherapy on patients’ mental health.

These findings are in line with Morykwas et al., who demonstrated in foundational studies that the physiological benefits of NPWT in animal models translate effectively into clinical outcomes, especially when combined with rehabilitative techniques [16,17]. Furthermore, integrating early rehabilitation with NPWT has been shown to significantly improve functional outcomes in patients with deep partial-thickness burns [18,19,20,21].

In a similar manner, the study by Zens et al. [22] found that NPWT demonstrated a significant advantage over standard wound therapy (SWT) for patients with wound healing by secondary intention. Their systematic review and meta-analysis, involving 48 studies and 4315 patients, highlighted that NPWT significantly enhanced wound closure (odds ratio 1.56, *p* = 0.008) and reduced hospital stays (mean difference−4.78 days, *p* = 0.005). Conversely, Jingchun Zhao and colleagues [23] conducted a retrospective study comparing the efficacy of polyhexamethylene biguanide and betaine (PHMB-B) versus normal saline as instillation solutions during NPWTi-d in diabetic foot infections. Their findings indicated no significant differences between the two solutions across several outcomes including wound bed preparation time, hospital stay, and overall cost, suggesting that both instillation solutions are equally effective. While Zens et al. demonstrated clear benefits of NPWT in general wound management, Zhao et al. pointed to the specific application of NPWTi-d, underlining that the choice of instillation solution might not significantly impact clinical outcomes in the context of diabetic foot infections. Both studies contribute to the nuanced understanding of NPWT’s utility and effectiveness in varying contexts and conditions.

Moreover, Webster et al. [24] and the study by Norman et al. explored the implications of NPWT on surgical wound healing by primary intention, with both studies concluding with nuanced outcomes. Webster et al. assessed the effects of NPWT on skin grafts and clean surgical wounds through a systematic review, involving five trials with 280 participants. They found no significant advantage of NPWT over standard dressings in enhancing wound healing, as adverse events were similar across both groups (risk ratio 0.97, CI 0.33 to 2.89). Interestingly, a separate trial within the study indicated a lower adverse event rate with a hospital-developed NPWT system compared to a commercial system, suggesting variability in the efficacy based on the type of NPWT system used. On the other hand, Norman et al. [25] conducted a comprehensive review involving 62 RCTs and over 13,000 participants, finding moderate-certainty evidence that NPWT likely reduces surgical site infections (SSIs) with a risk ratio of 0.73 (CI 0.63 to 0.85). However, their findings also indicated that NPWT had little impact on wound dehiscence and was not cost-effective for certain surgeries. Both studies underline the potential of NPWT to improve certain outcomes like reduced SSIs, yet they also highlight the complexity and variability of results depending on the wound type, NPWT system, and the economic implications of using such technology.

### 4.2. Study Limitations

An important limitation was the follow-up period, which varied from 10 weeks to 6 months. Although this duration provided relevant insights into the immediate and intermediate effects of the intervention, it may have been insufficient to fully evaluate the long-term impact of therapy on joint functionality and muscle strength. Field suggested that “extending follow-up periods to 12 months or more allows for the assessment of sustained therapeutic benefits and potential late-onset complications” [19].

Additionally, the absence of a control group without intervention represents a methodological limitation, as it does not allow for a direct comparison of the natural progression of untreated wounds. Variability in the application of physiotherapy techniques, even within a standardized protocol, could introduce measurement errors. Factors such as therapist experience, patient adherence to the protocol, and interpretational differences in applying techniques may affect the uniformity of clinical data. Finally, the specificity of the studied population—patients with acute wounds localized to the calf area—restricts the applicability of the results to other categories of patients, such as those with diabetic ulcers or chronic wounds.

## 5. Conclusions

The results of this study confirm that NPWT combined with specialized physiotherapy techniques leads to significant improvements in functional parameters in patients with post-traumatic and post-surgical wounds. Objective data analysis highlighted notable increases in joint mobility, with an improved range of motion in the ankle and knee, and a more pronounced reduction in edema in Group 2 compared to treatment with NPWT alone. Manual muscle testing also demonstrated a significant increase in muscle strength in the group that benefited from the combined intervention. These objective improvements were supported by rigorous statistical analyses, including significant correlations between functional variables and clinical indicators, demonstrating that integrating physiotherapy enhances the effects of NPWT. Furthermore, both short-term assessments and 6-month monitoring confirmed that the benefits obtained are sustained over time, highlighting the durability of the positive effects of the intervention. These findings provide strong evidence for clinicians regarding the adoption of personalized therapeutic strategies that emphasize optimizing functional parameters in the treatment of patients with acute wounds. Implementing an integrated protocol that combines NPWT and physiotherapy can significantly improve clinical recovery, reduce rehabilitation time, and enhance the quality of care provided.

## Figures and Tables

**Table 1:** Demographic and clinical characteristics of study patients.

Characteristics	NPWT (n = 110)	NPWT+FNP (n = 82)	*p*-Value	Cohen’s d
Age, years	37.86 ± 4.53	36.54 ± 5.50	0.069	0.28
Sex, men (%)	58 (52.7%)	47 (57.3%)	0.063	
Overweight (>25.0 kg/m^2^)	51 (46.36%)	54 (56.84%)	0.103	
Smoking	43 (39.1%)	31 (37.8%)	0.717	
CCI > 2	2 (1.81%)	2 (2.4%)	0.554	
Wound type			0.688	
- Open tibial fractures	41 (37.3%)	27 (32.9%)		
- Post-surgical wounds	47 (42.7%)	41 (50%)		
- Crush injuries	22 (20%)	14 (17.1%)		

**Table 2:** Ankle and knee mobility measurements at 10 days, 42 days, and 180 days between patients treated with NPWT exclusively and NPWT plus physiotherapy.

Variables	NPWT (n = 110)	NPWT+FNP (n = 82)	*p*-Value	Cohen’s d
Dorsiflexion (°)—10 days	10.05 ± 1.76	18.10 ± 1.63	<0.001	4.65
Plantar flexion (°)—10 days	29.58 ± 2.42	45.13 ± 2.56	<0.001	5.98
Knee flexion (°)—10 days	115.57 ± 5.32	134.58 ± 5.15	<0.001	3.50
Pronation (°)—10 days	11.40 ± 2.16	18.06 ± 2.12	<0.001	3.10
Supination (°)—10 days	20.22 ± 2.56	33.24 ± 2.65	<0.001	4.90
Dorsiflexion (°)—42 days	15.12 ± 1.73	23.10 ± 1.64	<0.001	4.75
Plantar flexion (°)—42 days	35.56 ± 2.48	54.05 ± 1.54	<0.001	6.73
Knee flexion (°)—42 days	130.01 ± 4.49	141.42 ± 3.74	<0.001	2.68
Pronation (°)—42 days	16.61 ± 1.59	18.06 ± 2.12	0.001	0.74
Supination (°)—42 days	27.90 ± 2.25	33.24 ± 2.65	0.980	0.01
Dorsiflexion (°)—180 days	18.15 ± 1.86	23.10 ± 1.64	<0.001	2.75
Plantar flexion (°)—180 days	44.60 ± 2.73	54.05 ± 1.54	<0.001	4.20
Knee flexion (°)—180 days	135.59 ± 4.52	141.42 ± 3.74	<0.001	1.40
Pronation (°)—180 days	18.11 ± 1.63	18.06 ± 2.12	0.838	0.03
Supination (°)—180 days	33.42 ± 2.31	33.24 ± 2.65	0.618	0.07

**Table 3:** Calf, edema circumference, and edema depth measurements at 10 days, 42 days, and 180 days between patients treated with NPWT exclusively and NPWT plus physiotherapy.

Variables	NPWT (n = 110)	NPWT+FNP (n = 82)	*p*-Value	Cohen’s d
Calf circumference (cm)—10 days	37.48 ± 1.84	37.06 ± 1.79	0.115	0.23
Edema circumference (cm)—10 days	41.15 ± 2.39	40.36 ± 2.32	0.023	0.33
Edema depth (mm)—10 days	1.49 ± 0.8	1.50 ± 0.69	0.957	0.01
Calf circumference (cm)—42 days	36.28 ± 1.8	36.39 ± 1.75	0.695	0.06
Edema circumference (cm)—42 days	37.49 ± 1.84	37.06 ± 1.80	0.115	0.23
Edema depth (mm)—42 days	0.9 ± 0.042	0.50 ± 0.23	<0.001	1.21
Calf circumference (cm)—180 days	35.23 ± 1.79	36.28 ± 1.76	<0.001	0.59
Edema circumference (cm)—180 days	36.29 ± 1.8	36.39 ± 1.75	0.695	0.06
Edema depth (mm)—180 days	0.53 ± 0.24	0.19 ± 0.14	<0.001	1.69

**Table 4:** MMT tibialis anterior and triceps surae measurements at 10 days, 42 days, and 180 days between patients treated with NPWT exclusively and NPWT plus physiotherapy.

Variables	NPWT (n = 110)	NPWT+FNP (n = 82)	*p*-Value	Cohen’s d
Initial MMT tibialis anterior	3.27 ± 0.67	3.08 ± 0.68	0.058	0.28
Initial MMT triceps surae	3.46 ± 0.67	3.18 ± 0.64	0.004	0.43
MMT tibialis anterior—10 days	3.60 ± 0.66	4.02 ± 0.69	<0.001	0.63
MMT triceps surae—10 days	3.88 ± 0.67	4.13± 0.63	0.007	0.38
MMT tibialis anterior—42 days	4.14 ± 0.60	4.69 ± 0.48	<0.001	1.02
MMT triceps surae—42 days	4.41 ± 0.53	4.82 ± 0.41	<0.001	0.86
MMT tibialis anterior—180 days	4.83 ± 0.37	4.96 ± 0.19	0.003	0.48
MMT triceps surae—180 days	4.95 ± 0.23	4.98 ± 0.16	0.303	0.16

**Table 5:** WHOQOL-BREF survey results.

Variables	NPWT (n = 110)	NPWT+FNP (n = 82)	*p*-Value	Cohen’s d
Physical domain—10 days	27.17 ± 5.83	39.30 ± 5.56	<0.001	2.15
Physical domain—42 days	55.52 ± 7.44	70.45 ± 6.88	<0.001	2.06
Physical domain—180 days	85.12 ± 5.41	94.53 ± 5.07	<0.001	1.77
Psychological domain—10 days	45.04 ± 6.14	54.78 ± 6.12	<0.001	1.61
Psychological domain—42 days	64.98 ± 5.96	74.13 ± 5.33	<0.001	1.63
Psychological domain—180 days	85.74 ± 5.37	95.06 ± 4.87	<0.001	1.83
Social domain—10 days	49.93 ± 6.11	59.21 ± 5.36	<0.001	1.64
Social domain—42 days	65.84 ± 7.03	77.02 ± 7.22	<0.001	1.59
Social domain—180 days	91.61 ± 5.05	97.66 ± 3.07	<0.001	1.44
Mean score—10 days	59.89 ± 5.86	69.24 ± 5.92	<0.001	1.59
Mean score—42 days	72.47 ± 6.86	82.02 ± 7.23	<0.001	1.37
Mean score—180 days	93.40 ± 3.95	98.99 ± 1.59	<0.001	1.67

**Table 6:** SF-36 survey results.

Variables	NPWT (n = 110)	NPWT+FNP (n = 82)	*p*-Value	Cohen’s d
SF-36 score—10 days	40.54 ± 5.78	49.40 ± 6.26	<0.001	1.51
SF-36 score—42 days	61.44 ± 7.2	72.69 ± 7.06	<0.001	1.59
SF-36 score—180 days	85.60 ± 5.35	94.21 ± 5.19	<0.001	1.77

**Table 7:** HADS survey results.

Variables	NPWT (n = 110)	NPWT+FNP (n = 82)	*p*-Value	Cohen’s d
Anxiety score—10 days	13.14 ± 2.39	10.64 ± 2.38	<0.001	1.05
Anxiety score—42 days	7.93 ± 1.89	5.13 ± 1.83	<0.001	1.48
Anxiety score—180 days	4.44 ± 1.35	2.08 ± 1.43	<0.001	1.68
Depression score—10 days	10.87 ± 2.31	8.36 ± 2.30	<0.001	1.09
Depression score—42 days	6.95 ± 1.89	4.32 ± 1.70	<0.001	1.43
Depression score—180 days	3.74 ± 1.42	1.33 ± 1.27	<0.001	1.66

**Table 8:** VAS-scale for pain survey results.

Variables	NPWT (n = 110)	NPWT+FNP (n = 82)	*p*-Value	Cohen’s d
VAS score—10 days	6.7 ± 1.05	5.52 ± 1.01	<0.001	1.14
VAS score—42 days	3.31 ± 1.34	1.35 ± 1.03	<0.001	1.63
VAS score—180 days	0.05 ± 0.31	0 ± 0	0.137	0.19

**Table 9:** Correlations over 10 days, 6 weeks, and 6 months post intervention.

Time	Variable	Correlated Outcome	Coefficient (r)	*p*-Value
10 Days	MMT tibialis anterior	SF-36	0.090	0.214
10 Days	MMT triceps surae	SF-36	0.012	0.870
10 Days	MMT tibialis anterior	WHOQOL-BREF	0.162	0.025
10 Days	MMT triceps surae	WHOQOL-BREF	0.056	0.438
10 Days	MMT tibialis anterior	HADS Anxiety	−0.089	0.221
10 Days	MMT triceps surae	HADS Anxiety	−0.113	0.117
10 Days	MMT tibialis anterior	VAS	−0.203	0.005
10 Days	MMT triceps surae	VAS	−0.138	0.056
10 Days	Pronation	SF-36	−0.248	0.001
10 Days	Pronation	WHOQOL-BREF	0.517	<0.001
10 Days	Pronation	HADS Anxiety	−0.381	<0.001
10 Days	Pronation	VAS	−0.460	<0.001
10 Days	Supination	SF-36	0.560	<0.001
10 Days	Supination	WHOQOL-BREF	0.596	<0.001
10 Days	Supination	HADS Anxiety	−0.450	<0.001
10 Days	Supination	VAS	−0.476	<0.001
10 Days	Edema circumference	SF-36	−0.011	0.879
10 Days	Edema circumference	WHOQOL-BREF	−0.049	0.497
10 Days	Edema circumference	HADS Anxiety	0.070	0.334
10 Days	Edema circumference	VAS	0.165	0.023
6 Weeks	MMT tibialis anterior	SF-36	0.231	0.001
6 Weeks	MMT triceps surae	SF-36	0.195	0.007
6 Weeks	MMT tibialis anterior	WHOQOL-BREF	0.218	0.002
6 Weeks	MMT triceps surae	WHOQOL-BREF	0.207	0.004
6 Weeks	MMT tibialis anterior	HADS Anxiety	−0.210	0.004
6 Weeks	MMT triceps surae	HADS Anxiety	−0.170	0.019
6 Weeks	MMT tibialis anterior	VAS	−0.303	<0.001
6 Weeks	MMT triceps surae	VAS	−0.260	<0.001
6 Weeks	Pronation	SF-36	0.355	<0.001
6 Weeks	Pronation	WHOQOL-BREF	0.093	0.198
6 Weeks	Pronation	HADS Anxiety	−0.310	<0.001
6 Weeks	Pronation	VAS	−0.365	<0.001
6 Weeks	Supination	SF-36	0.451	<0.001
6 Weeks	Supination	WHOQOL-BREF	0.429	<0.001
6 Weeks	Supination	HADS Anxiety	−0.451	<0.001
6 Weeks	Supination	VAS	−0.448	<0.001
6 Weeks	Edema circumference	SF-36	−0.179	0.013
6 Weeks	Edema circumference	WHOQOL-BREF	−0.076	0.295
6 Weeks	Edema circumference	HADS Anxiety	0.153	0.034
6 Weeks	Edema circumference	VAS	0.168	0.020
6 Months	MMT tibialis anterior	SF-36	0.205	0.004
6 Months	MMT triceps surae	SF-36	0.032	0.656
6 Months	MMT tibialis anterior	WHOQOL-BREF	0.076	0.297
6 Months	MMT triceps surae	WHOQOL-BREF	−0.010	0.887
6 Months	MMT tibialis anterior	HADS Anxiety	−0.117	0.105
6 Months	MMT triceps surae	HADS Anxiety	0.017	0.820
6 Months	MMT tibialis anterior	VAS	0.036	0.619
6 Months	MMT triceps surae	VAS	0.026	0.720
6 Months	Pronation	SF-36	−0.017	0.814
6 Months	Pronation	WHOQOL-BREF	0.044	0.543
6 Months	Pronation	HADS Anxiety	0.073	0.312
6 Months	Pronation	VAS	−0.024	0.738
6 Months	Supination	SF-36	−0.044	0.544
6 Months	Supination	WHOQOL-BREF	−0.053	0.461
6 Months	Supination	HADS Anxiety	−0.036	0.621
6 Months	Supination	VAS	−0.086	0.238
6 Months	Edema circumference	SF-36	0.057	0.435
6 Months	Edema circumference	WHOQOL-BREF	0.051	0.486
6 Months	Edema circumference	HADS Anxiety	−0.088	0.226
6 Months	Edema circumference	VAS	0.084	0.249

## Data Availability

Data availability is subject to hospital approval.
